# Fragmentation of an In-Date Copper Intrauterine Device: An Unusual Complication

**DOI:** 10.7759/cureus.73344

**Published:** 2024-11-09

**Authors:** Johnbosco Mamah, Thokozani Dube, Ejiro Aramabi, Emmanuel Unwaha, Max Dagba

**Affiliations:** 1 Obstetrics and Gynaecology, East Suffolk and North Essex NHS Foundation Trust, Colchester, GBR

**Keywords:** contraceptive device complications, copper iud, copper iud disintegration, copper iud fragmentation, intrauterine device

## Abstract

We present a rare case involving a 25-year-old woman who had fragmentation of a copper intrauterine contraceptive device (IUD) while still within its recommended lifespan. The patient's symptoms included lower abdominal pain, offensive vaginal discharge, and the passage of copper IUD fragments. The pelvic examination confirmed the presence of the copper IUD strings, and with her consent, the device was removed. Upon inspection, it was discovered that one horizontal arm of the copper IUD had no copper coils, and the second horizontal arm was missing half of its copper coil. The device was sent for microbial culture. The patient's symptoms promptly resolved with conservative management. This case raises awareness about the potential for copper IUD disintegration, even when the device is in-date. Sexual and reproductive health providers should consider this complication when discussing the pros and cons of a copper IUD with intended users.

## Introduction

The copper intrauterine contraceptive device (IUD) is one of the most widely used methods of long-acting reversible contraception, with a reported effectiveness of over 99%; it has a failure rate of 0.8% for typical use and 0.6% for perfect use within the first year of insertion [1-3]. Copper IUDs, such as the TCu380A, are designed to remain effective for up to 10-12 years by releasing copper ions, which create a hostile environment for the sperm, the ovum, and the embryo [1]. Copper in the cervical mucus also inhibits the passage of sperm into the upper reproductive tract [1,2]. 

An ideal candidate for a copper IUD should have no evidence of a pelvic infection, have a regular sexual partner as it does not prevent sexually transmitted diseases, and be able to recognize symptoms of complications related to the IUD [1]. According to the United Kingdom Medical Eligibility Criteria for Contraceptive Use (UKMEC), an IUD is categorized as UKMEC 1 for nulliparous women aged 20 or more [1]. This means it can be used under any condition. If a copper IUD is not appropriate, alternative options include implants, intrauterine systems, and injectables [1].

Copper IUDs are not usually affected by drugs or enzymes, hence their well-documented safety and durability [1-5]. However, complications may arise, including device expulsion, malposition, and, in rare instances, disintegration or fragmentation of the device [2-12]. Device fragmentation, particularly within the lifespan of an IUD, is an unusual occurrence with limited literature. Disintegration of the copper components may lead to symptoms such as abnormal uterine bleeding, pelvic pain, and, in more severe cases, migration of fragments into the uterine wall or adjacent structures [3-7]. The exact mechanism for the premature fragmentation of copper IUDs remains unclear. However, factors such as material fatigue, manufacturer error, mechanical stress, or patient-specific uterine conditions may play a role [1-5]. Some reports have suggested a possible association of *Actinomyces* infection with copper IUD fragmentation. *Actinomyces* species are known to cause pelvic infections and may contribute to the breakdown of the copper components of the IUD [3,4].

We report a case of a copper IUD fragmentation within five years of insertion, well in advance of the expiration date, in a patient who presented with pelvic discomfort and abnormal vaginal discharge. This case underscores the importance of thorough patient counseling and obtaining informed consent before inserting a copper IUD. Providers should be aware of this possible complication, examine copper IUDs for completeness after removal, and discuss the potential risks and benefits with their patients. Overall, the copper IUD remains a safe and effective IUD [1,8-12].

## Case presentation

**Figure 1:**
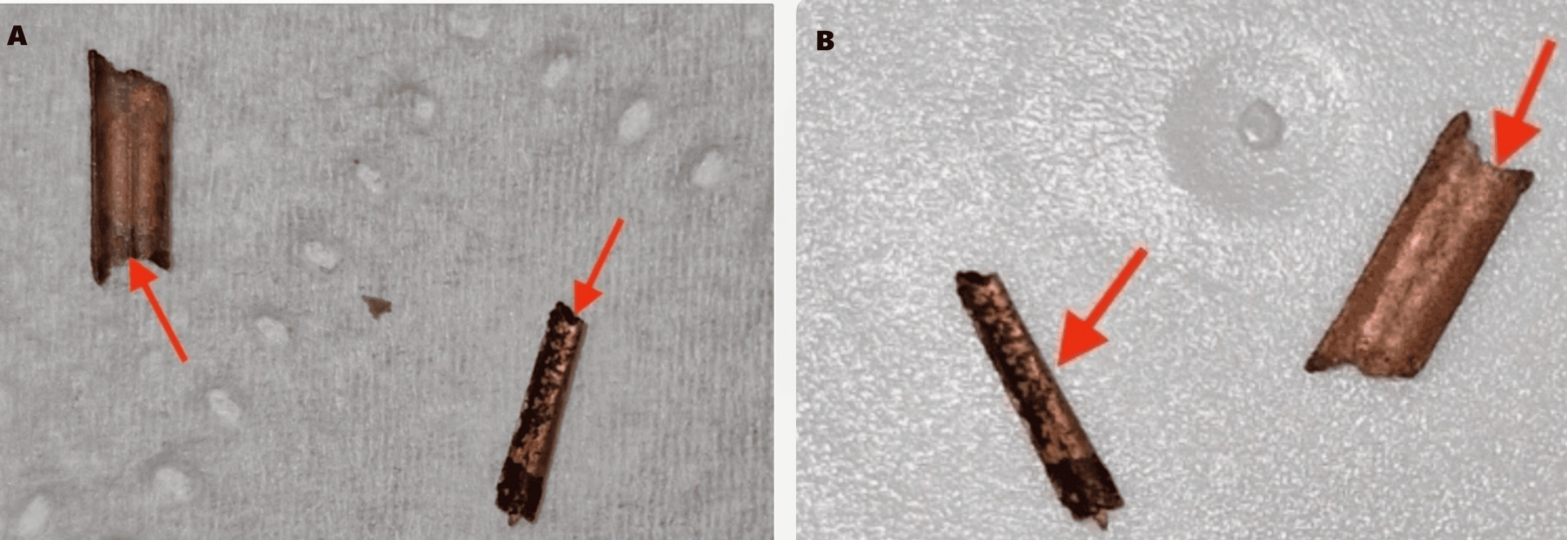
Part of the copper intrauterine device fragments passed (A) Pieces of copper intrauterine device coils passed per vaginam by the patient in the days leading up to her presentation. (B) Pieces of copper intrauterine device coils passed by the patient on the eve of her presentation in our unit. The red arrows point to the remnants of the broken parts of the copper coil.

A 25-year-old woman, gravida 0, para 0, presented to the acute gynecology unit of the East Suffolk and North Essex National Health Service Foundation Trust with complaints of worsening chronic lower abdominal pain and abnormal vaginal discharge for which she had a course of antibiotics for bacterial vaginosis. Her symptoms got worse a few weeks before her presentation, and about one week before the day of her presentation, she felt a sharp pain in the perineum and passed some copper IUD fragments per vaginam (Figure 1).

**Figure 2:**
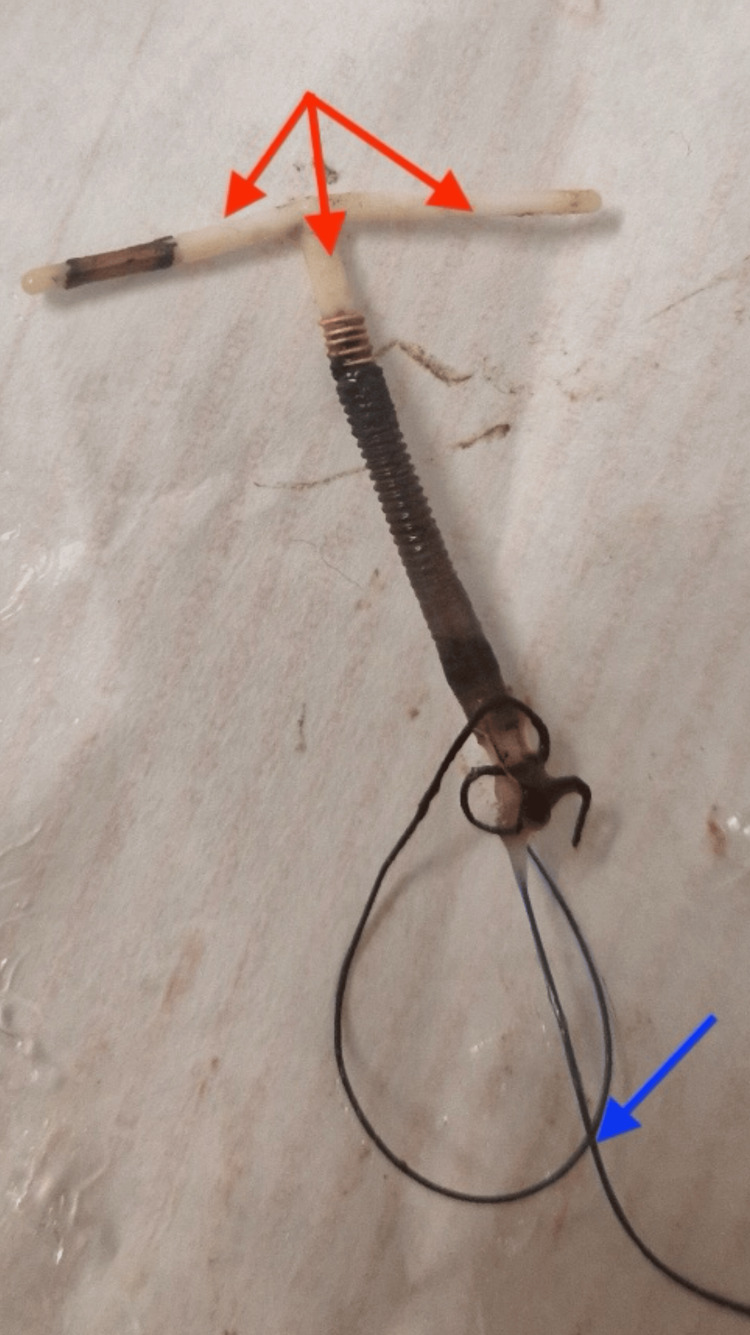
Image of the removed copper intrauterine device The copper IUD after removal. The red arrows indicate the horizontal arms that had lost their copper coils and the vertical arms partly missing their copper coils. The IUD string depicted by the blue arrow appears intact and is used to extract the device

According to her health records, she had an in-date (unexpired) TCu380A copper IUD inserted five years earlier, with no complications during or after insertion. The device was within its recommended lifespan of 12 years. She had no previous history of sexually transmitted infection. She had an irregular menstrual period and a negative urine pregnancy test on presentation. The physical examination revealed mild tenderness in the lower abdomen, and the pelvic exam with a Cusco speculum revealed that the copper IUD strings were at the external cervical os. Given her symptoms and having passed some copper fragments, it was agreed that the copper IUD be removed; this was done without complications. The removed device was examined, and we observed missing copper fragments on its horizontal arms and partly on the vertical arms (Figure 2).

The copper IUD was sent for microbiological culture and sensitivity. We requested an urgent pelvic X-ray to investigate for retained copper fragments, but it was negative. Still worried about possible retained copper fragments, the patient subsequently had a transvaginal ultrasound and a pelvic computed tomography scan, which showed no retained copper fragments. The microbial culture results of the copper IUD showed colonization of the copper IUD with *Actinomyces* species; at this time, the patient’s symptoms had resolved, and she did not require further treatment based on expert advice from a microbiologist, and this is also consistent with the United Kingdom Faculty of Sexual and Reproductive Healthcare (FSRH) advice for *Actinomyces *colonization of IUDs in the absence of evidence of a pelvic infection. 

## Discussion

This case reports the occurrence of a rare complication of a copper IUD while still within its expected lifespan of 10-12 years. The exact cause of this device fragmentation is unclear. Several factors could contribute to the premature disintegration of a copper IUD [2-12]. One potential cause is material fatigue due to mechanical stress [4-9]. The uterus is a dynamic organ that undergoes constant changes, including uterine contractions and hormonal fluctuations, which may exert stress on the IUD [8,9]. Over time, repeated uterine contractions could weaken the device's structure, particularly at stress concentration points, such as the arms or copper wiring. While copper IUDs undergo rigorous testing before market approval, any sporadic manufacturing defects in the device’s design or manufacturing process will enhance the likelihood of its disintegration [9,10]. Inflammation and corrosion of the copper components are other plausible explanations for the disintegration, as copper IUDs rely on the slow release of copper ions to prevent pregnancy. The copper IUD is built to be corrosion-resistant, but prolonged exposure to the slightly alkaline environment of the uterus may accelerate its degradation; the uterine environment causes the copper to undergo oxidative dissolution, which results in the release of cupric ions; over time, the cupric ions are oxidized to cupric oxide, which forms a thick substance that coats the copper IUD and may occasionally break down due to repeated cycles of this chemical reaction leading to corrosion [5-8,11,12].

In rare cases, undiagnosed or subclinical infections could lead to an exaggerated inflammatory response, compromising the device's integrity [3-6]. There have been reports of an association between copper coil colonization with *Actinomyces* and subsequent copper IUD disintegration [3,4]. This is theorized to result from the chronic inflammatory process triggered by *Actinomyces *that ultimately leads to the disintegration of the copper IUD [3,4,10]. *Actinomyces* are considered commensals in healthy individuals but can also be opportunistic organisms; they are prevalent in the female genital tract due to microtissue trauma from the IUD, which leads to an anaerobic milieu that makes the organism thrive [3,4,10]. *Actinomyces *form biofilms on the copper coil, making it difficult to eradicate and, in the long term, leading to enhanced copper coil degradation and disintegration [3,4,11,12]. While it is logical to offer antibiotics for confirmed pelvic infection with *Actinomyces*, colonization does not indicate the presence of a disease. Women are not routinely screened for *Actinomyces*, and management is individualized in cases with an incidental finding of *Actinomyces* copper IUD colonization [1]. The FRSH recommends the treatment of *Actinomyces *if there is evidence of pelvic infection [1]. Educating patients to recognize pelvic infection symptoms and seeking medical attention as needed is essential.

The clinical presentation of IUD disintegration can be variable and may not be recognized by the patient or the clinician. Symptoms such as abnormal uterine bleeding, abnormal vaginal discharge, or pelvic discomfort are common and may be attributed to other gynecological causes [1-6]. Passage of copper fragments is confirmatory but will rely on the patient’s awareness and recognition. The clinician must maintain a high index of suspicion when patients present with unusual symptoms, even if the device is within its approved duration of use [6-12]. The management of a disintegrated IUD involves prompt removal to prevent further complications, such as uterine perforation, chronic inflammation, or migration of fragments into the surrounding structures [7-12]. Imaging studies should be conducted to identify and locate radio-opaque retained copper fragments [4-7]. Hysteroscopic removal is the preferred approach, allowing for the precise localization and retrieval of IUD fragments within the uterine cavity [7,13]. Some experts have reported ultrasound-guided extraction using grasping forceps [8]. Once removed, an uncomplicated course is expected. Mental health support should be provided to the patient as this could be very stressful and a source of significant anxiety. Going forward, more research is needed to better understand the material properties of copper IUDs, the factors contributing to their premature degradation, and whether specific patient populations are more prone to this complication. 

## Conclusions

This case report underscores the importance of patient education on recognizing abnormal symptoms of copper IUD fragmentation and the need to seek timely medical evaluation. On the part of the practitioner, it highlights the need to recognize that unusual complications may exist with the use of the copper IUD, and these should be discussed with the potential user to enable informed decision-making.

Further research is required to understand the mechanisms underlying early IUD degradation and identify ways to mitigate this occurrence.
